# The disease burden associated with *Campylobacter* spp. in Germany, 2014

**DOI:** 10.1371/journal.pone.0216867

**Published:** 2019-05-15

**Authors:** Julia Lackner, Michael Weiss, Christine Müller-Graf, Matthias Greiner

**Affiliations:** Department of Exposure, Federal Institute for Risk Assessment, Berlin, Germany; University of Alberta Department of Resource Economics and Environmental Sociology, CANADA

## Abstract

Bacteria of the genus *Campylobacter* are an important cause of human illness worldwide. Campylobacter infections are expressed as gastroenteritis and can lead to severe sequelae like reactive arthritis, Guillain-Barré syndrome, irritable bowel syndrome and inflammatory bowel disease. In Germany, *Campylobacter*-associated gastroenteritis cases are notifiable but there is no reporting obligation for the sequelaes and the disease burden is clearly underestimated. The aim of our study was to quantify reliably the current disease burden of all *Campylobacter* spp.-associated diseases for Germany with the method of disability-adjusted life years (DALYs). DALYs combine mortality and morbidity in a single summary measure, whereby one DALY represents the loss of one year in full health. For acute gastroenteritis, we estimated 967 DALYs of which only 484 DALYs were detected within the reporting system. Overall, we estimated that 8811 DALYs were caused by the campylobacter-related diseases known so far. 98% of the DALYs were associated with morbidity and 2% with mortality. Mortality was caused by the health outcomes Gastroenteritis and Guillain-Barré syndrome exclusively.

## Introduction

Foodborne diseases cause significant morbidity and mortality and are a growing public health problem worldwide. Bacteria of the genus *Campylobacter* spp. are one of the most common causes of gastroenteritis (GE) in Germany and other European countries [1]. Occasionally, infections with *Campylobacter* spp. lead to sequelae like reactive arthritis (reA), Guillain-Barré syndrome (GBS), irritable bowel syndrome (IBS) and inflammatory bowel disease (IBD) [2]. In Germany, only gastroenteritis cases are notifiable and the incidence increased from 65.98 per 100,000 population (2001) to 85.53 per 100,000 population (2015) [3]. As a result of the missing reporting obligations for the sequelae [4] and a general underestimation of the gastroenteritis cases (e.g. no visit of general practitioner, no stool sample test), the actually reported cases represent only a small part of the disease burden associated with *Campylobacter* spp. in reality [5].

Disability-adjusted life years (DALYs) are a commonly used summary measure to quantify the burden of a disease, injury or risk factor. DALYs combine mortality and morbidity into a common metric and allow for comparisons over time periods or between countries [6, 7]. For some countries, the disease burden of *Campylobacter* spp. has already been estimated with the DALY method [8–12]. Until now, however, only one pilot study based on DALYs, was conducted for Germany. In this study, the disease burden of the years 2003 to 2005 was calculated by using the mean number of reported *Campylobacter*-associated gastroenteritis cases. Additionally to the endpoint GE the sequelae reA, GBS and IBD were included [11].

The aim of our study was to quantify the current disease burden of all *Campylobacter* associated diseases known so far in Germany and to develop a model, which can in future be used for disease burden estimation caused by *Campylobacter spp*.

Therefore, we combined the DALY method with a preceding Monte Carlo simulation in which we integrated published information on reported gastroenteritis cases, underreporting factors, and probabilities of sequelae. This resulted in an improved estimation of most likely values for *Campylobacter*-associated incidences and corresponding confidence intervals. This information was used subsequently for the disease burden calculation. Finally, we compared our results with results of disease burden calculation for Germany, which would be based on reported gastroenteritis cases exclusively and the results of the pilot study.

## Methods

The reported campylobacteriosis cases [3, 13] and associated deaths [14] for Germany in 2014 were included as fixed values in the disease burden calculation. The incidence and mortality of mild GE and the sequelae were estimated by Monte Carlo simulation with the parameters for the respective distributions obtained from literature (more details see below under “Gastroenteritis”). Simulations were conducted by using the mc2d package in R (Version 3.4.1, r-project) with 10,000 iterations [15, 16]. Afterwards, for each age- and sex-class the minimum, most likely and maximum values of these distributions were used to create beta-PERT-distributions as incidence input variable for the DALY calculator [12, 13].

### DALY calculation

The health outcomes caused by *Campylobacter* spp. were summarized in DALYs, following the methodology proposed by Murray and colleagues [6, 7]:

DALY=YLD+YLL

The Years Lived with a Disability or Disease (YLD) measures the morbidity. The calculation is based on the incidence (*n*) of a health outcome (*l*) multiplied by the duration (*t*) and the disability weight (*w*) of a specific illness [17]:

$$YLD=\sum_{l}n_{l}\times t_{l}\times w_{l}$$

The Years of Life Lost due to mortality (YLL) measures the premature mortality. The calculation is based on the number of all fatal cases (*d*) due to the specific health outcome (*l*) multiplied by the remaining life expectancy in years (*e*) [17]:

$$YLL=\sum_{l}d_{l}\times e_{l}$$

The DALY calculation was performed with the package “DALY” [16, 18] in R (Version 3.4.1, r-project). DALY calculation based on 100,000 iterations.

### Population

We estimated DALYs with reference to the German population in the year 2014 (81,197,537 inhabitants [19]) and considered five age classes (0–4 years, 5–14 years, 15–44 years, 45–59 years, over 60 years). The life expectancy was derived from the life-table 2012/2014 reported by the German Federal Statistic Office, female and male life expectancy was set to 83.05 and 78.13 years, respectively [20].

### Health outcomes

**Fig 1:**
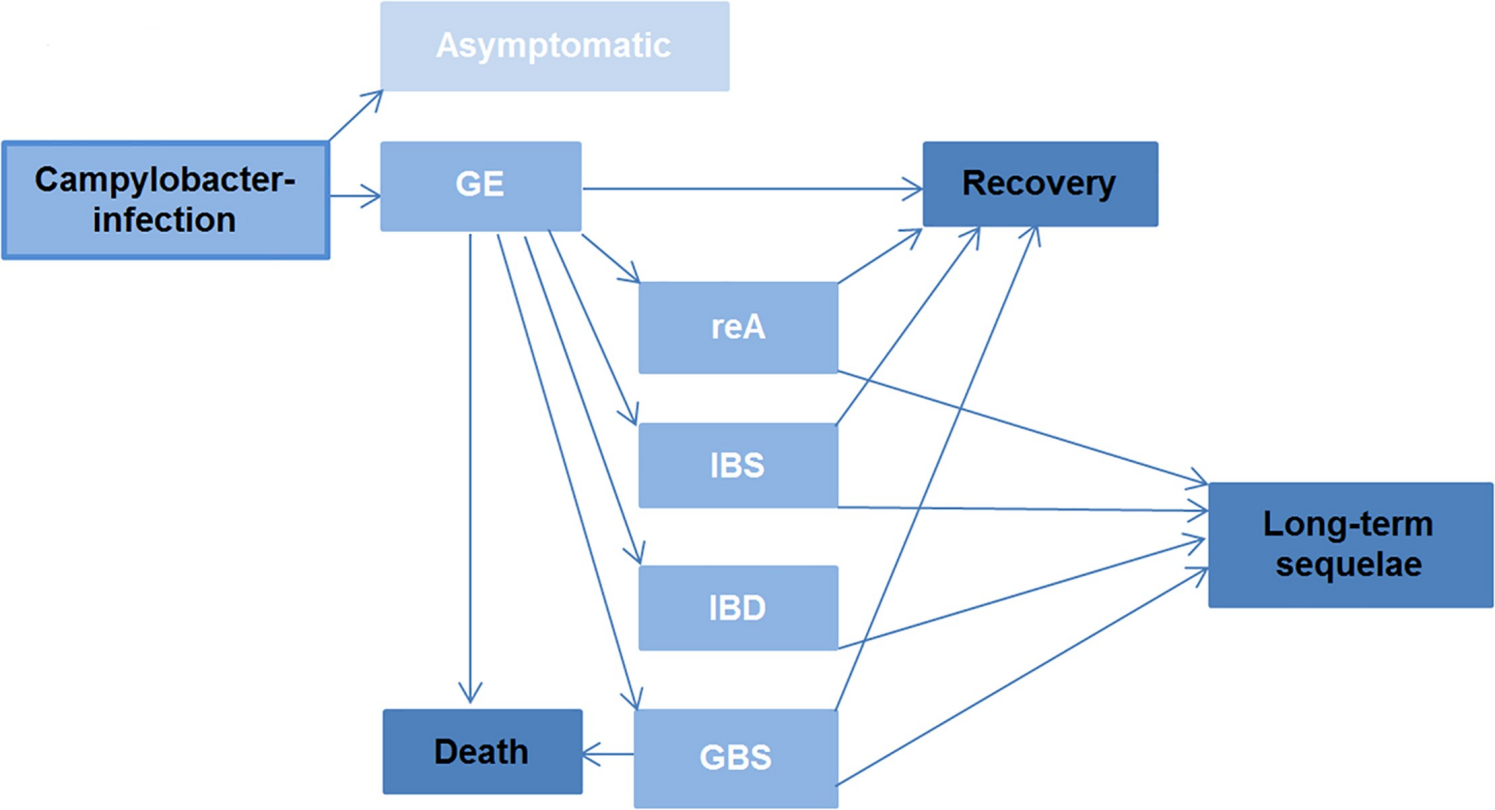
Illustration of the outcome tree of campylobacter infections (slightly modified version of Pires (2014) [21]). GBS = Guillain Barré syndrome, GE = Gastroenteritis, IBD = inflammatory bowel disease, IBS = irritable bowel syndrome, reA = reactive arthritis.

We determined the health outcomes of campylobacter infections by a literature review and identified eight health endpoints: The symptomatic GE with the severities mild, moderate and severe and four sequelae (reactive arthritis (reA), mild and severe Guillain-Barré syndrome (GBS), irritable bowel syndrome (IBS) and inflammatory bowel disease (IBD)). Fig 1 shows the outcome tree, which was adopted from Pires (2014) [21] with the main difference that we changed the prognosis of IBD, because our literature review showed that IBD is always a lifelong disease (Fig 1) [22].

The effect of parameter uncertainty and underreporting (mild gastroenteritis) following the idea of a sensitivity analyses can be estimated from Table 1 in the supporting information.

**Table 1:** Parameters used in the pyramid reconstruction model for *Campylobacter* spp. in Germany, 2014 (according to Haagsma et al. (2012) [5]).

Symbol	Description	Distribution
***Country-specific parameters***		
**Probability of visiting a GP with (in Germany)**		
***a***	Bloody diarrhoe	Beta (22;21)
***b***	Non-bloody diarrhoea	Beta (459; 885)
**Probability of submitting a stool sample for a consulting patient with:**		
***c***	Bloody diarrhoe	Beta (11; 11)
***d***	Non-bloody diarrhoea	Beta (156; 302)
***e***	Probability of submitting a stool sample for a hospitalized patient	Beta (28; 8)
**Probability of analysing *Campylobacter* spp. in samples for**		
***f***	Patients visiting a GP	Beta (9.9; 0.1)
***g***	Hospitalized patients	Beta (9.9; 0.1)
**Probability of reporting a positive laboratory result for:**		
***h***	Patients visiting a GP	Beta (9.9; 0.1)
***i***	Hospitalized patients	Beta (9.9; 0.1)
***Pathogen-specific parameters***		
***j***	Sensitivity of laboratory analysis for *Campylobacter* spp.	Triang (0.7; 0.76; 0.82)
***k***	Proportion of bloody diarrhoea in population cases	Beta (4.74; 21.3)

GP = General Practitioner

### Gastroenteritis

In various disease burden studies GE-cases were divided into three classes with different severity levels [12, 23, 24]. We followed this grouping and defined mild cases as unreported cases, moderate cases as cases with visit to a general practitioner and severe cases as cases resulting in a hospital stay. As incidence of severe cases, the reported numbers of age- and sex-specific cases with hospitalization were used and as incidence of moderate cases, we used the difference of all reported cases and severe cases as defined above [3, 13]. To estimate the incidence of mild cases, we reconstructed the surveillance pyramid for Campylobacter infections in Germany according to Haagsma et al. (2012) [5] by using the data of the year 2014. Table 1 shows the country- and pathogen-specific parameters used in the pyramid reconstruction model and Table 2 describes the model, which leads to a multiplier [5]. To calculate the incidence of mild GE-cases, we calculated for each sex and age group a multiplier, which was multiplied by the reported numbers of moderate GE [3].

**Table 2:** Model for reconstructing the surveillance pyramid for *Campylobacter* spp. in Germany, 2014 (according to Haagsma et al. (2012) [5]).

Symbol	Description	Distribution
***nr***	Number of reported cases per year	Data for each age and gender group [3]
***nh***	Number of hospitalized cases per year	Data for each age and gender group [13]
***nGP***	Number of cases who are not hospitalized, but visit a GP	*n*_*R*_–*n*_*H*_
***p***	Probability of visiting a GP with gastroenteritis	k*a+(1−*k*)*b*
***m***	Probability of submitting a stool sample when visiting a GP	k*c+(1−*k*)*d*
**Probability of reporting a case for**		
***n***	Patients visiting a GP	m*f*j*h
***o***	Hospitalized patients	e*g*j*i
***NGP***	Total number of cases visiting a GP	*n*_*G*_/*n*
***NH***	Total number of hospitalized cases	*n*_*H*_/*o*
***NP***	Total cases in the population	(*N*_*GP*_+*N*_*H*_)/*p*
***NGP-***	Cases in the population who do not visit a GP	*Np*−(*N*_*GP*_+*N*_*H*_)
***M***	Multiplier	*Np*/*n*_*R*_

GP = General Practitioner

Furthermore, we used reported age- and sex-specific mortality data to calculate the YLL. Altogether, nine *Campylobacter*–associated deaths with an average age at death of 78 years were reported [14].

We assumed that the duration of a GE is the same in both sexes, but can vary with age. For babies and toddlers, we used 4, 6 and 8 days as durations for mild, moderate and severe diseases according to the estimations of the Foodborne Disease Burden Epidemiology Reference Group (FERG) [23], whereas for the other age classes, we used durations of 3, 10 and 14 days [25].

The severity of the three forms of GE was estimated without differentiation into age and gender classes following a European study of disease weights [26]. In order to account for uncertainty, beta-PERT distributions were generated using the confidence intervals reported in this study [26].

### Reactive arthritis

ReA as a sequelae of GE occurs only very rarely in childhood, so we assumed that children under 15 years do not get this disease [27, 28].

For adolescents and adults, we translated probabilities of reA occurrence after GE into beta-PERT distributions reflecting the incidences [27] (Table 3). Since reA is a potentially life-long illness, the duration was fitted by using an exponential function to allow for short and long durations. We used an exponential function with a mean disease duration of 0.608 years for all age- and sex-classes which provides a good coverage of the reported reA durations [25].

**Table 3:** Summary of the parameters that were used to fit beta-PERT distributions reflecting the probabilities to develop sequelae after a *Campylobacter*-associated GE.

Health outcome	Most likely value	Minimum	Maximum	Reference
**Reactive arthritis**	7.5%	0.6%	24%	[27]
**Guillain Barré syndrom**	0.07%	0%	2.2%	[2, 31]
**Irritable bowel syndrom**	8.8%	0.03%	16.7%	[2, 31]
**Inflammatory bowel disease**	0.4%	0.12%	0.62%	[2]
**Guillain Barré syndrom mortality**	4.1%	2.4%	6%	[17]

Disease weights were parameterized by using a beta-PERT distribution with the lowest value found in the literature (0.023) as minimum [24] and 0,21 and 0,37 as most likely and maximum value. Both parameters have already been used in various disease burden studies [11, 21, 25, 29, 30].

### Guillain-Barré syndrome

To estimate how often GBS occurs after a GE, two recent systematic reviews were screened [2, 31]. As parameters of a beta-PERT distribution, we used as minimum and maximum the lowest and highest probabilities of the individual studies reported in the reviews and as most likely value the result of a meta-analysis (0.07%) [2, 31] (Table 3). Samples of these distributions were multiplied by the probability of developing a mild (17%) or severe (83%) GBS [17]. The age and sex-distribution of the GBS based on the German hospitalization data for the year 2014, because we assumed that any form of GBS was treated in a hospital [13].

Based on our literature review, we fitted an exponential distribution for the duration of the mild GBS with an estimated mean duration of 0.417 years [32–35] and defined the severe GBS as a life-long disease [17].

The GBS is often divided into five severity grades using an F-score and for each severity grade exists a disability weight [36]. We used for our classification a uniform distribution of the disability weights for F1 (0.044) and F2 (0.137) for the mild GBS and a beta-PERT distribution for the severe (F3 as minimum (0.215), F4 as most likely value (0.367), F5 as maximum (0.46)) [37].

The severe GBS is a potentially fatal health outcome. To estimate the mortality, we fitted a beta-PERT distribution with the probabilities reported in Mangen et al. (2013) [17]. To determine the average age at death caused by severe GBS, the age distribution of all reported fatalities caused by GBS for Germany in 2014 was adopted [14].

### Irritable bowel syndrome

For the occurrence probability of an IBS after a Campylobacter infection, we used again the reported minimum and maximum values of the two systematic reviews mentioned above to fit a beta-PERT distribution [2, 31] (Table 3). As the most likely value, we used a probability of 8.8% which is commonly used in various disease burden studies [12, 17, 21, 38, 39].

The duration of IBS was described by using an exponential function. According to literature, half of the patients recover after 5–6 years. Therefore, we fitted the exponential function with a mean duration of 5.5 years for all age- and sex classes [40, 41].

The beta-PERT distribution of the IBS disability weight for all ages- and sex-classes was based on the confidence intervals (2.5% CI 0.05, 97.5% 0.077) and the mean value (0.062) of the European disability weight study [26].

### Inflammatory bowel disease

The probability of developing an IBD following a campylobacter infection was estimated based on literature data. As with the other health endpoints, we generated a beta-PERT distribution based on the reported probabilities. Two studies (reviewed in [2]) reported separate probabilities for the occurrence of ulcerative colitis and the Crohn's disease after *Campylobacter*-associated GE. Since both diseases are a type of IBD, we added these probabilities. The probabilities, which were used for all age- and sex classes, are listed in Table 3.

In accordance with other disease burden studies, we assumed that IBD is a life-long disease [21, 24, 29, 30].

As with the health endpoint IBS, the values of the European disability weight study were used to fit a beta-PERT distribution of the IBD severity. Thereby, we used as minimum 0.184, as most likely value 0.221 and as maximum 0.26 [26].

## Results

We estimated that in Germany in the year 2014 805,029 persons were affected by a *Campylobacter*-associated GE and 13,468 persons developed a sequelae (estimates are the mean values of the simulations). In addition to the nine reported deaths caused by GE, we estimated three more deaths caused by GBS.

**Fig 2:**
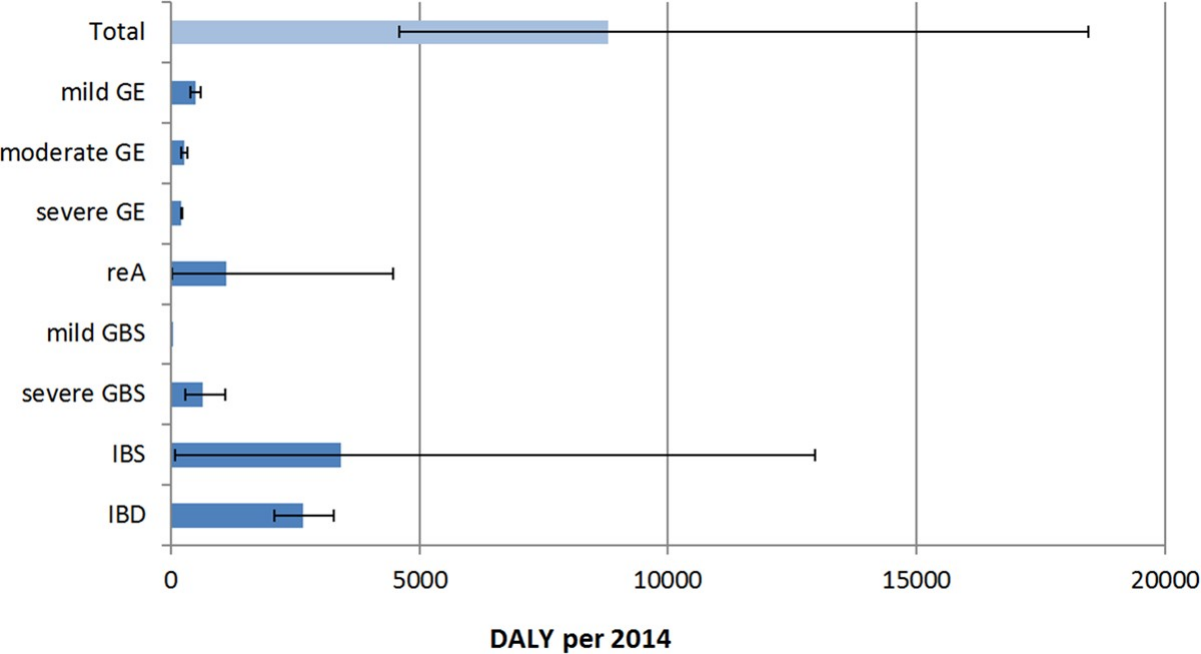
Mean values and 95% CI of the campylobacter-related disease burden according to the health endpoints for 2014. GBS = Guillain Barré syndrome, IBD = inflammatory bowel disease, IBS = irritable bowel syndrome, reA = reactive arthritis.

Overall, we estimated that an average of 8811 DALYs (95% confidence interval: 4603–18,468 DALYs) were caused by Campylobacter infections in the year 2014 (Fig 2). This corresponds to 10.85 DALYs per 100,000 inhabitants. 2% of the DALYs were associated with mortality and 98% with morbidity.

Fig 2 shows the calculated means of disease burden for the eight outcomes. To illustrate the uncertainty of the estimation, the 95% CI of the endpoints were plotted. The confidence intervals of the reA and IBS are very wide, whereas the severities of the three forms of GE have narrow intervals. Also, the overall burden of disease has a wide range due to the uncertainties in the estimation of the sequelae, which are mostly severe and persistent. In the Appendix, the results for all eight health endpoints are presented in detail.

With 3422 DALYs IBS was the strongest contributor to the total burden of campylobacteriosis, whereas GBS was the weakest (643 DALYs). Nevertheless, GBS contributed to 45% of the disease burden due to mortality (71 DALYs). The remaining YLLs were caused by severe gastroenteritis (88 DALYs).

**Fig 3:**
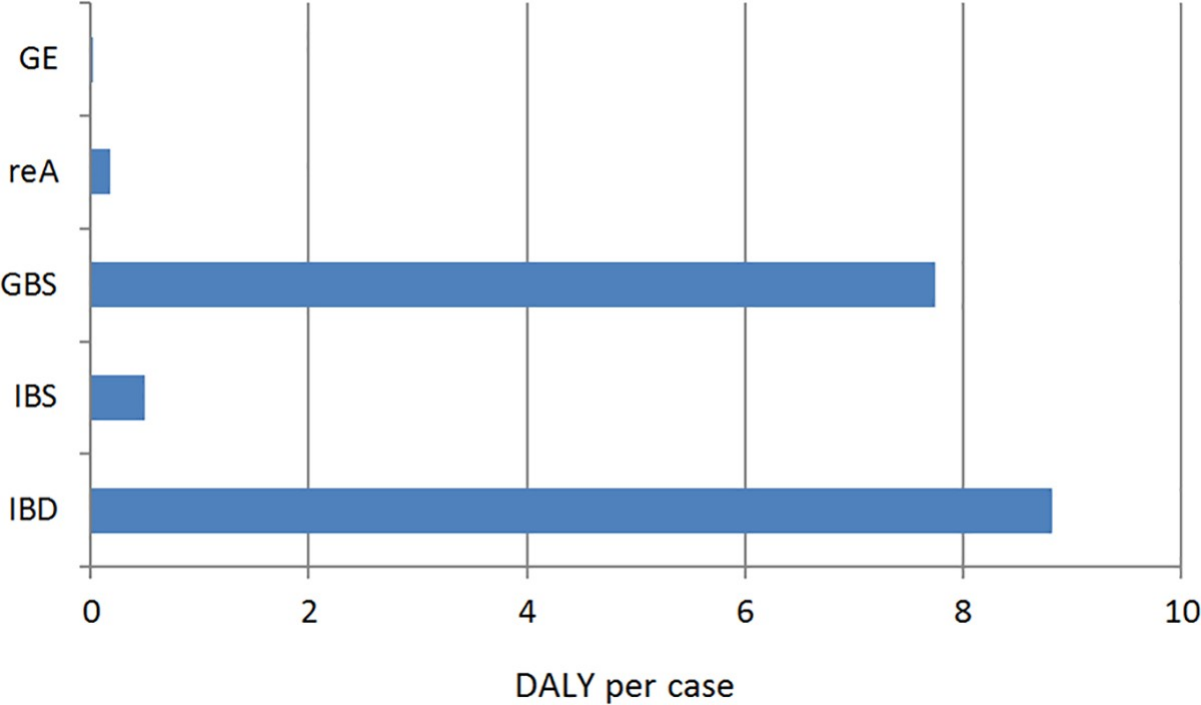
Disease burden of the sequelae per case. GBS = Guillain Barré syndrome, IBD = inflammatory bowel disease, IBS = irritable bowel syndrome, reA = reactive arthritis.

We estimated, that the lowest disease burden per case was caused by the GE (0.001 DALY per case) and the highest burden was estimated for the sequelae GBS (7.747 DALY per case) and IBD (8.817 DALY per case) (Fig 3).

**Fig 4:**
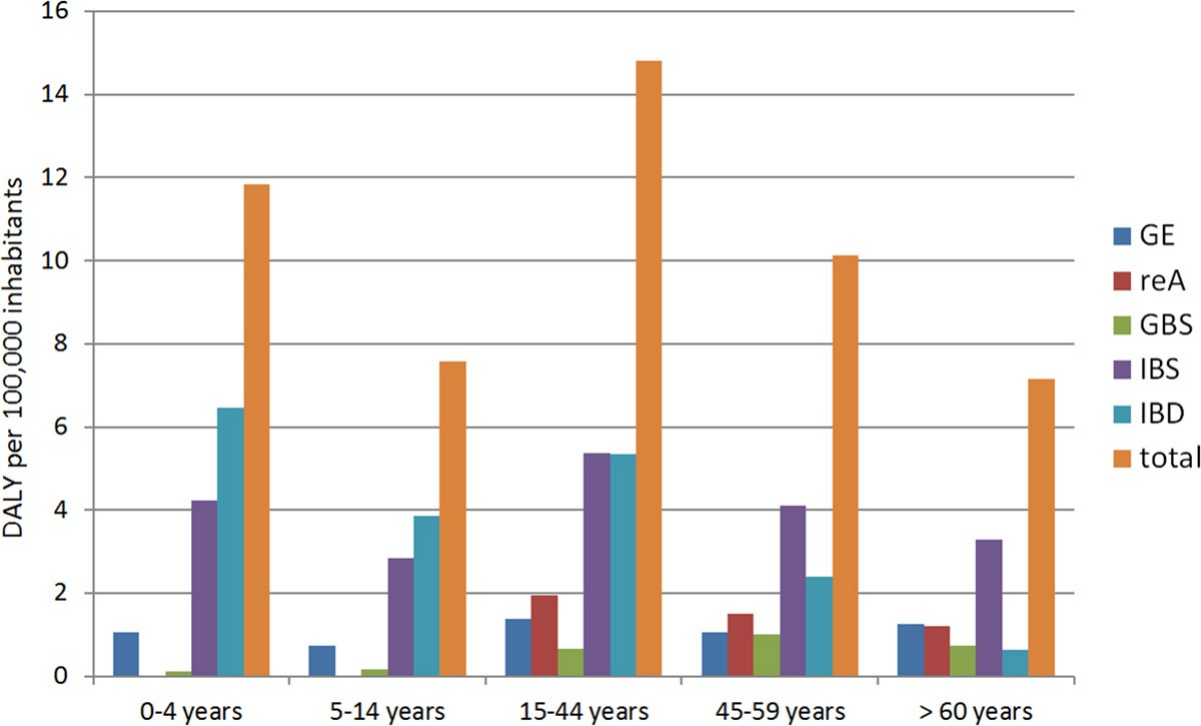
Comparison of the disease burden of the health endpoints per age group. GBS = Guillain Barré syndrome, IBD = inflammatory bowel disease, IBS = irritable bowel syndrome, reA = reactive arthritis. For comparison, reported campylobacteriosis incidence per age group (per 100,000 population): 0–4 years: ♂ **=** 104, ♀ = 85; 5–14 years: ♂ **=** 71, ♀ = 55; 15–44 years: ♂ **=** 122, ♀ = 120; 45–59 years: ♂ **=** 99, ♀ = 86; >60 years: ♂ **=** 83, ♀ = 66.

With regard to the disease burden age-distribution, we discovered that the sequelae reA and GBS are not relevant in the childhood. IBS and IBD contributed the most to the disease burden of children and adults younger than 60 years. In adults older than 60 years the contribution of IBD decreased (Fig 4).

**Fig 5:**
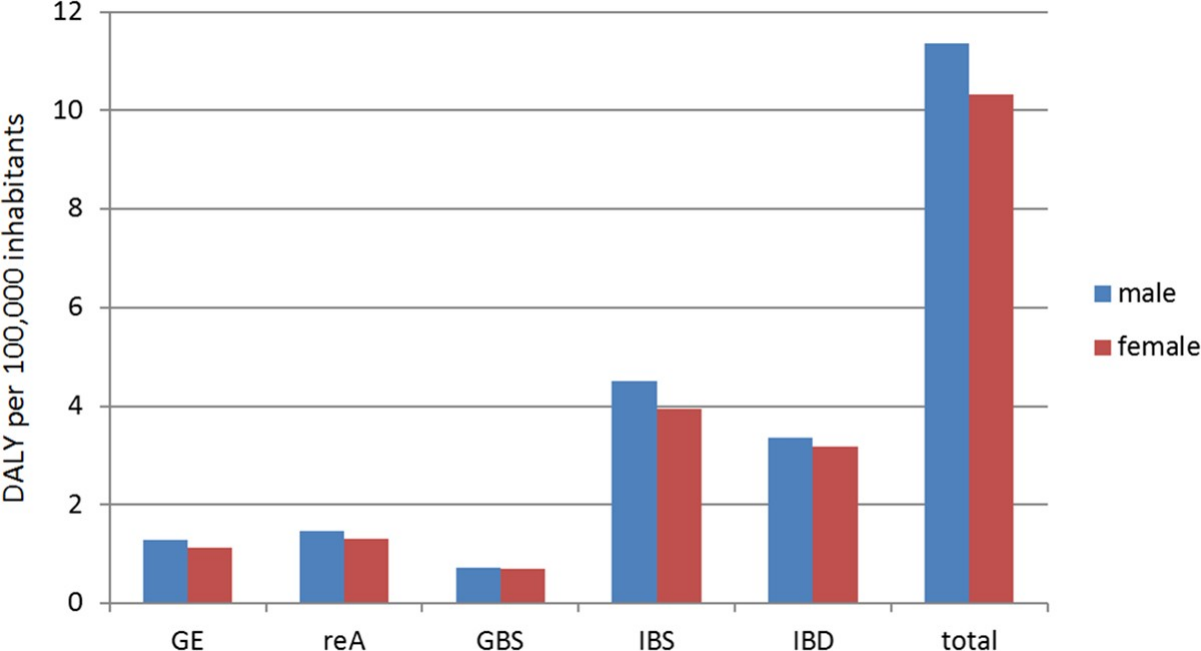
Sex-specific analysis for the different health outcomes and the total disease burden.

Sex-specific analysis showed that the overall disease burden of campylobacter was about 6% lower in women than in men. No differences were found for GBS and IBD, whereas the men were more severely affected by GE and the sequelae reA and IBS (Fig 5).

Overall, 8811 DALYs were estimated by campylobacter-related diseases. Of these, 967 DALYs were attributed to an acute GE, whereas the remaining disease burden was caused by sequelae. Of the burden of disease by a GE, only 484 DALYs were attributed to reported cases. Therefore, it can be concluded, that probably only 5% of the overall *Campylobacter*-associated disease burden can be derived from reporting data.

## Discussion

In this study, we calculated the disease burden of the *Campylobacter* spp.-associated diseases GE, reA, GBS, IBS and IBD based on a mixture of reporting, hospitalization and mortality data and incidences that were estimated based on literature data for the year 2014 in Germany. A huge majority of the disease burden was attributed to morbidity (98%) compared to mortality. This finding is consistent with all other international published studies, which subdivided the diseases burden of *Campylobacter* spp. into YLLs and YLDs [9–12, 21, 23, 24, 30, 36, 37, 42, 43]. In these studies, the estimated mortality varied between 0% [42] and 31% [11] of the total disease burden. This variance is mainly a result of the decision on which sequelae were included in the study. Toljander et al. (2012) included only reA and GBS in their estimation. The chronic and non-fatal diseases IBS and IBD, which usually lead to a high morbidity, were not considered. In addition, Toljander et al. (2012) estimated that depending on the scenario 29% respectively 40% of the burden of GBS persists of YLL [11]. In our study, we estimated a lower proportion of YLLs and attributed only 11% of the burden of GBS to premature mortality.

For a sound comparison of the disease burden between different countries or time periods, it is essential that the same health endpoints are used in the underlying studies. In older disease burden calculations for the Netherlands, in which the burden of IBS was not considered, the *Campylobacter* spp.-associated disease burden was estimated to lie between 7.4 and 8.3 DALYs per 100,000 inhabitants [122, 178], whereas recent results, which include IBS, range from 18.5 DALYs to 21.6 DALYs per 100,000 inhabitants [60, 118, 129]. In our study (IBS included), a considerably lower result of 10.85 DALYs per 100,000 inhabitants was estimated for Germany. Besides of country-specific variability in food production and consumption behavior or differences in the natural abundance of *Campylobacter* spp., differences in the methods applied in the burden estimation are also likely reasons for these differences. Havelaar et al. (2012) estimated all incidences (except those of IBD) to be higher than in our study (e.g. 8000 versus 6886 incidences of IBS) [24], although the total number of inhabitants in Germany is almost five times higher as in the Netherlands [44].This deviation is mainly based on the used GE data, which are the basis for the incidence estimation of the sequelae. The estimated incidences of GE in the Netherlands based on a Dutch community-based cohort study performed from 1998 through 1999 and a nested case-control study to identify the proportion of cases with and without consultation of a general practitioner, whereas in our study reported cases of the year 2014 were used and additionally an estimation of the underreported cases was conducted [3, 5, 24].

Besides the Dutch studies, there is another Danish study, which focused on the same health endpoints (GE, GBS, reA, IBS, IBD) as we do in our study and estimated a disease burden of 28,4 DALYs per 100,000 inhabitants [21]. The Danish study determined the IBS incidences with a relatively narrow beta-PERT distribution, whereas our study used a much broader beta-PERT based on recent reviews [2, 31].

When comparing proportions of the health endpoints, it becomes clear that in our estimation the IBD contributes with 30% noticeable more to the overall disease burden than in the Danish study (about 11%). This difference can be explained by the fact that all other health endpoints were attributed to a higher burden of disease in the Danish study, whereas the disease burden of campylobacter-induced IBD were estimated in both studies with similar incidences, disability weights and the same disease duration [21].

Considering the age distribution in the Danish study, it can be seen that the largest disease burden occurs in the 0–4 year-old age group, which is followed by the burden in adulthood [124]. In our study, the highest burden of disease occurs in young adults (15–44 year olds) and the second highest in the 0–4 year-old age group. The difference can be explained by the use of age-specific incidences for the sequelae reA and GBS in our study. Thus, reA and GBS are very rare diseases in the childhood, which therefore only lead to a low burden of disease.

Regarding sex specific differences, both studies found a higher campylobacter-related disease burden in males, whereby the differences between the sexes in the Danish study were larger. Since the burden of disease has been calculated for men and women with the same disease severity and duration, this discrepancy is caused by the differences in the reported gender-specific incidences [124].

A pilot study estimated a disease burden of 6165.1 DALYs for the health outcomes GE, reA, GBS and IBD based on reported data for Germany in 2003–2005 [42]. This disease burden is lower than the calculated burden in our study, because we included one health endpoint (IBS) more. Considering the disease burden in our study without the health endpoint IBS, the estimated burden is with 5389 DALYs lower than in the pilot study. With the knowledge, that the incidence of Campylobacteriosis cases increased and that unreported cases are included in our study this result is unexpected. This discrepancy occurred mainly due to methodological differences: In the pilot study a disability weight of the GE (0,393) was used, that is considerable higher than that we used in our study [42]. In our study, the highest disability weight was used in the beta-PERT-distribution for the severe GE, where we used as the most likely value 0.239, as minimum 0.202 and as maximum 0.285.

Another important difference between our study and the pilot study is that in the pilot study a value to describe the probability to develop a reA after a GE was used, that is about twice as high [42].

The long-term goal of estimating disease burden is to rank the various non-infectious and infectious agents or pathogens. This ranking will identify those pathogens that have the largest effect on the health of populations and helps to develop appropriate public health policies measures to prevent and reduce the burden in the population. So far, the burden of disease has been calculated only for a few pathogens for Germany [42, 45, 46]. Based on these few studies, we developed two rankings, which are attached in the appendix. It can be determined that the highest disease burden per year is caused by methylmercury (14,186 DALYs) and HIV (21,397 DALYs) [42, 46]. The exposure to methylmercury can lead to an IQ reduction in unborn children, which can result in a lifelong mental retardation [46]. HIV is also a lifelong and serious illness, which often leads to premature death [42]. Two studies looked on the burden of influenza and salmonellosis and come to different results [42, 45]. Plass et al. (2014) calculated 19,115 DALYs for salmonellosis and 33,116 DALYs for influenza per year, whereas van Lier and Havelaar (2007) reported 4248 DALYs for salmonellosis and 2162 DALYs for influenza per year [42, 45]. These divergent results can be explained by the use of different methods for DALY estimation. Plass et al. (2014) used a multiplication factor to include the unreported cases which leads to a higher disease burden on the population level, whereas van Lier and Havelaar (2007) used reported cases exclusively [42, 45]. The results of our study also include unreported cases, but are lower than the estimated disease burden of salmonellosis. This difference might be a result of how the burden of mortality was estimated. Plass et al. (2014) calculated 7418 DALYs per year due to premature deaths caused by an acute salmonellosis, whereas we estimated 88 DALYs due to premature deaths caused by an acute campylobacteriosis [45]. This estimation is in line with reported causes of deaths from 1998–2015 in Germany, which recorded more deaths due to an acute salmonellosis (12–36 deaths per year) than due to an acute campylobacteriosis (0–10 deaths per year) [47]. It can be concluded that the disease burden of salmonellosis is higher than the burden of campylobacteriosis when the unreported cases are included.

Plass et al. (2014) and Havelaar and Lier (2007) estimated the lowest disease burden for measles in Germany (234–740 DALYs per year). Measles is a viral disease that can lead to serious, partially fatal, complications (otitis media, pneumonia, encephalitis, subacute sclerosing panencephalitis) [42, 45]. The low disease burden is not an expression of the severity of the disease, but shows that a low measles incidence in Germany was achieved by vaccinations, which reduces the burden of disease at the population level. At the individual level, both studies estimated a burden of disease of 0.3 DALYs per case [42, 45]. The severity of the measles disease can be shown, compared with our estimated campylobacter-related disease burden of 0.01 per case. Despite the low campylobacter-associated burden of disease per case, this study has shown that the disease burden at population level is important and the reliable burden is substantial underestimated, if only the reporting data of the acute GE were used for the estimation.

Different studies estimated that the foodborne transmission route of *Camplyobacter* spp. is the most important route with an attribution between 42–82% [10, 24, 29, 38, 48]. Targeted disease prevention is necessary to reduce the *Campylobacter*-associated disease burden. To inform the population about kitchen hygiene mistakes and cross-contaminations may be an important starting point. Furthermore, other transmission routes (e.g. the contamination in the environment, contact with farm animals and traveling) should increasingly be in the focus of research to develop preventive measures for risk groups.

## Supporting information

S1 Table Results of the overall disease burden and the health endpoints separately. (DOCX)

S1 Fig Camp = campylobacteriosis, EHEC = enterohaemorrhagic *Escherichia coli*, Hep B = hepatitis B, HIV = human immunodeficiency virus, Influ = influenza, Meas = Measles, MeHg = methylmercury, Salm = salmonellosis, Tub = tuberculosis. (TIF)

S2 Fig Camp = campylobacteriosis, EHEC = enterohaemorrhagic *Escherichia coli*, Hep B = hepatitis B, HIV = human immunodeficiency virus, Influ = influenza, Meas = Measles, MeHg = methylmercury, Salm = salmonellosis, Tub = tuberculosis. (TIF)
