# Supplementary material for: The disease burden associated with Campylobacter spp. in Germany, 2014
Source: PLoS One. 2019 May 15;14(5):e0216867. doi: 10.1371/journal.pone.0216867 (PMC6519833; doi:10.1371/journal.pone.0216867)
Supplement: S1 Table — (DOCX) [file pone.0216867.s001.docx]

|  | Mean | Median | 2.5% Percentile | 97.5% Percentile |
| --- | --- | --- | --- | --- |
| Overall disease burden | | | | |
| DALY | 8811 | 7828 | 4603 | 18468 |
| YLD | 8652 | 7669 | 4442 | 18303 |
| YLL | 160 | 157 | 115 | 222 |
| Cases | 818,092 | 816,988 | 722,284 | 920,343 |
| Deaths | 12 | 12 | 10 | 15 |
| Mild gastroenteritis | | | | |
| DALY | 492 | 490 | 405 | 592 |
| YLD | 492 | 490 | 405 | 592 |
| YLL | 0 | 0 | 0 | 0 |
| Cases | 727,855 | 726,766 | 631,739 | 830,121 |
| Deaths | 0 | 0 | 0 | 0 |
| Moderate gastroenteritis | | | | |
| DALY | 268 | 264 | 214 | 340 |
| YLD | 268 | 264 | 214 | 340 |
| YLL | 0 | 0 | 0 | 0 |
| Cases | 63,244 | 63,244 | 63,244 | 63,244 |
| Deaths | 0 | 0 | 0 | 0 |
| Severe gastroenteritis | | | | |
| DALY | 216 | 216 | 203 | 230 |
| YLD | 128 | 128 | 115 | 141 |
| YLL | 88 | 88 | 88 | 88 |
| Cases | 13,930 | 13,930 | 13,930 | 13,930 |
| Deaths | 9 | 9 | 9 | 9 |
| Reactive arthritis | | | | |
| DALY | 1118 | 724 | 25 | 4468 |
| YLD | 1118 | 724 | 25 | 4468 |
| YLL | 0 | 0 | 0 | 0 |
| Cases | 6198 | 6165 | 4072 | 8538 |
| Deaths | 0 | 0 | 0 | 0 |
| Mild Guillain Barré syndrome | | | | |
| DALY | 1 | 0 | 0 | 3 |
| YLD | 1 | 0 | 0 | 3 |
| YLL | 0 | 0 | 0 | 0 |
| Cases | 14 | 14 | 6 | 26 |
| Deaths | 0 | 0 | 0 | 0 |
| Severe Guillain Barré syndrome | | | | |
| DALY | 642 | 623 | 299 | 1095 |
| YLD | 571 | 552 | 232 | 1020 |
| YLL | 71 | 68 | 26 | 133 |
| Cases | 69 | 66 | 27 | 125 |
| Deaths | 3 | 3 | 1 | 6 |
| Irritable bowel syndrome | | | | |
| DALY | 3422 | 2331 | 85 | 12965 |
| YLD | 3422 | 2331 | 85 | 12965 |
| YLL | 0 | 0 | 0 | 0 |
| Cases | 6886 | 6892 | 5260 | 8493 |
| Deaths | 0 | 0 | 0 | 0 |
| Inflammatory bowel disease | | | | |
| DALY | 2654 | 2643 | 2087 | 3277 |
| YLD | 2654 | 2643 | 2087 | 3277 |
| YLL | 0 | 0 | 0 | 0 |
| Cases | 301 | 301 | 253 | 347 |
| Deaths | 0 | 0 | 0 | 0 |
