# Supplementary figures and images for: The disease burden associated with Campylobacter spp. in Germany, 2014
Source: PLoS One. 2019 May 15;14(5):e0216867. doi: 10.1371/journal.pone.0216867 (PMC6519833; doi:10.1371/journal.pone.0216867)

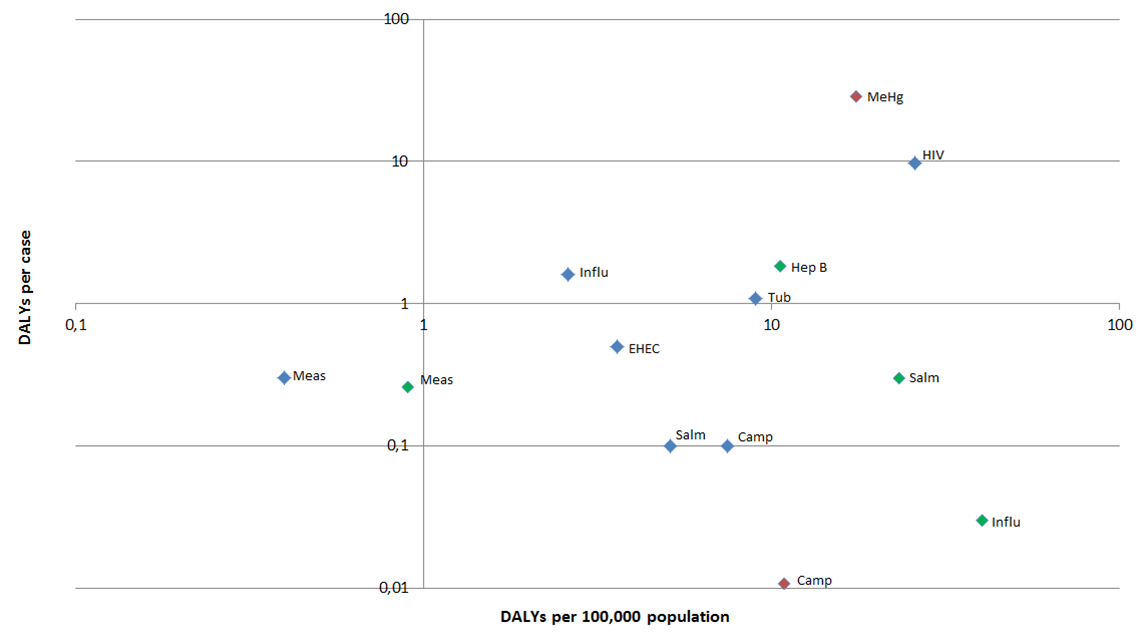

Supplement: S1 Fig — (TIF) [file pone.0216867.s002.tif]

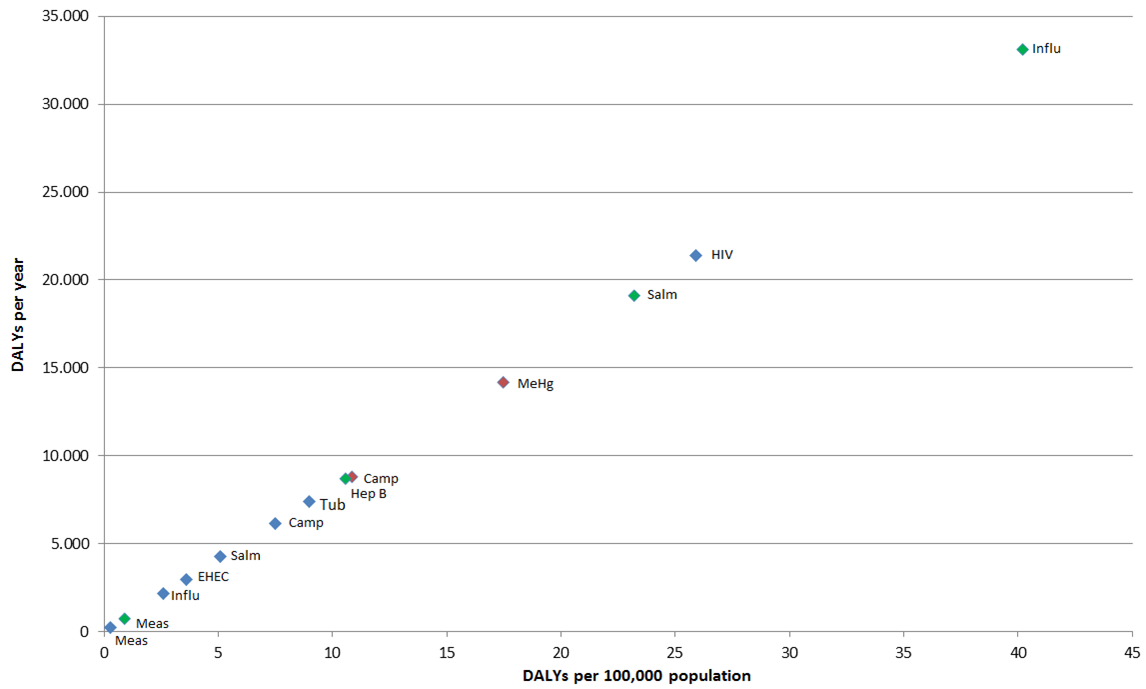

Supplement: S2 Fig — (TIF) [file pone.0216867.s003.tif]
